# Relationship Between 1,5 Anhydroglucitol, Glycemia, and Breastfeeding During Pregnancy and Postpartum: A Pilot Study

**DOI:** 10.1210/jendso/bvae207

**Published:** 2024-11-22

**Authors:** Marti D Soffer, Kaitlyn E James, Michael Callahan, Emily A Rosenberg, William H Barth, Camille E Powe

**Affiliations:** Harvard Medical School, Boston, MA; Division of Maternal Fetal Medicine, Department of Obstetrics, Gynecology, and Reproductive Biology, Massachusetts General Hospital, Boston, MA 02114, USA; Harvard Medical School, Boston, MA; Department of Obstetrics, Gynecology, and Reproductive Biology, Massachusetts General Hospital, Boston, MA 02114, USA; Deborah Kelly Center for Outcomes Research, Massachusetts General Hospital, Boston, MA 02114, USA; Diabetes Unit, Massachusetts General Hospital, Boston, MA 02114, USA; Harvard Medical School, Boston, MA; Division of Endocrinology, Diabetes, and Metabolic Diseases, Medical University of South Carolina, Charleston, SC 29425, USA; Harvard Medical School, Boston, MA; Division of Maternal Fetal Medicine, Department of Obstetrics, Gynecology, and Reproductive Biology, Massachusetts General Hospital, Boston, MA 02114, USA; Harvard Medical School, Boston, MA; Department of Obstetrics, Gynecology, and Reproductive Biology, Massachusetts General Hospital, Boston, MA 02114, USA; Diabetes Unit, Massachusetts General Hospital, Boston, MA 02114, USA; Division of Endocrinology, Diabetes, and Hypertension, Brigham and Women's Hospital, Boston, MA 02114, USA

**Keywords:** gestational diabetes, gestational glycemia, postpartum screening, postpartum diabetes, 1,5 anhydroglucitol, pregnancy biomarkers, breastfeeding

## Abstract

**Background:**

Assessments for hyperglycemia are vital to pregnancy and postpartum (PP) care, but gold-standard oral glucose tolerance tests (OGTTs) are burdensome. We examined changes in 1,5 anhydroglucitol (1,5AG) levels during gestation and PP and assessed for associations with other measures of glycemia.

**Study Design:**

Pregnant participants (n = 50) in the Study of Pregnancy Regulation of Insulin and Glucose cohort underwent OGTTs at a mean of 13 weeks ([visit 1 (V1)] and 26 weeks [visit 2 (V2)] of gestation and PP. Nonpregnant controls had a single OGTT. 1,5AG was measured using frozen plasma samples. Changes in 1,5AG across pregnancy were assessed with longitudinal mixed effects linear models. We assessed relationships between 1,5AG and glycemia at each timepoint using Spearman correlations and linear regression models. To determine the relationship of 1,5AG with breastfeeding (BF) status, stratified analyses were performed.

**Results:**

1,5AG decreased from V1 to V2 (β = −3.6 μg/mL, *P* < .001) and remained low PP compared to V1 (β = −1.4 μg/mL, *P* = .018). Comparisons between pregnant/PP and nonpregnant participants revealed lower 1,5AG values at all timepoints (V1 β = −9.9μg/mL, *P* < .001; V2 β = −14.0 μg/mL, *P* < .001, PP β = −11.4μg/mL, *P* < .001). There was no association between 1,5AG and glycemia. Compared to those exclusively feeding formula, 1,5AG levels were significantly lower in exclusively BF women (β = −8.8 μg/mL, *P* < .001) and intermediate in women feeding both breastmilk and formula (β = −6.1μg/mL, *P* < .001), independent of glycemia.

**Conclusion:**

1,5AG decreases during gestation and remains low PP. Breastfeeding is associated with lower 1,5AG levels, indicating plausible excretion into breastmilk. 1,5AG is unlikely to be useful in assessing glycemia in pregnant or PP women.

Gestational diabetes mellitus (GDM) is a common pregnancy complication, affecting more than 1 in 20 pregnancies in the United States [1-3]. Screening of all pregnant patients for GDM with oral glucose tolerance tests (OGTTs) is standard prenatal care given the known maternal and neonatal benefits of identification and treatment of GDM [4, 5]. Though some of the pathophysiology of the disease is related to hormonally mediated changes in the placenta, GDM has implications that extend beyond pregnancy: GDM is associated with an up to 70% risk of progression to diabetes in subsequent decades [6-8]. Given this risk of progression, it is recommended for patients with recent GDM to undergo OGTT screening at 4 to 12 weeks postpartum (PP) to determine the persistence or absence of dysglycemia [6, 9].

OGTT testing is cumbersome and associated with low rates of test completion [7, 10-15]; therefore, other glycemic biomarkers have been studied. Despite its promise and use in the nonpregnant population, the relationship between hemoglobin A1c (A1c) and glycemia is altered in pregnancy due to changes in red blood cell kinetics [16]. Regarding the PP population, while some studies have shown A1c to be useful in conjunction with fasting blood glucose, the biomarker has not demonstrated sufficient evidence to replace the OGTT in the PP screening of patients who had pregnancies complicated by GDM [16-20]. In the laboratory, OGTTs can also be affected by in vitro glycolysis, which can introduce measurement error if samples are not placed on ice or processed shortly after collection [21, 22].

1,5 anhydroglucitol (1,5AG) is an inert biomarker unaffected by fasting status or in vitro glycolysis [23]. Outside of pregnancy, low levels of 1,5AG correlate well with hyperglycemia in the preceding 48 hours to 2 weeks [24-26]. 1,5AG is obtained as part of a normal diet and, in times of normoglycemia, remains at a steady state due to an overall low concentration and without undergoing metabolism [27]. 1,5AG is known to compete with glucose at the level of the renal tubule, and given the preferential reabsorption of glucose, 1,5AG is excreted in the urine during times of hyperglycemia [24, 28]. While some prior researchers have suggested some correlation between 1,5AG and OGTT values during pregnancy and PP, most studies were small, were cross-sectional, or only included people with diabetes [29-31]. A few other studies have evaluated the levels of 1,5AG among PP women; however, they have been limited by the lack of data on glycemia or diet, both of which may interact with the serum level of 1,5AG as well as its excretion [30, 32]. We know of no prior studies that have analyzed PP maternal 1,5 AG levels in relation to breastfeeding (BF) status.

We sought to characterize changes in the levels of 1,5AG in a pilot study designed to better examine the relationship between this biomarker and glycemia during pregnancy and PP, while accounting for glycemia, diet, and lactation.

## Materials and Methods

In this pilot study, we evaluated a randomly selected group of participants who were part of the Study of Pregnancy Regulation of INsulin and Glucose cohort. This initial study sought to evaluate changes in insulin physiology in pregnancies with euglycemia as well as with GDM [33]. Participants were recruited both through an academic medical center as well as through advertisements in the surrounding area. Pregnant women in the first trimester were enrolled and were longitudinally monitored throughout pregnancy and PP. Nonpregnant women were enrolled as part of a cross-sectional study. People were eligible for the parent study if they had at least 1 risk factor for GDM [ie, history of prior pregnancy affected by GDM, first-degree family history of diabetes (DM) or GDM, or body mass index (BMI) ≥ 25 kg/m^2^ plus 1e additional risk factor] [34]. Participants were excluded if they had pregestational DM or were using medications known to affect glucose tolerance (ie, metformin or systemic corticosteroids). Those who went on to develop GDM were included in the pilot study. The Institutional Review Board of our institution approved the study, and all participants provided written informed consent.

Participants were studied 3 times across pregnancy: at 7 to 15 weeks gestation [visit 1 (V1)], in mid- to late pregnancy at 24 to 30 weeks gestation [visit 2 (V2)], and at 6 to 24 weeks PP [visit 3 (V3)]. For the purposes of this pilot analysis, a subset of 50 participants who attended all 3 study visits and had complete data available were selected at random. Baseline demographic information was obtained via participant survey, which included self-reported age, gravidity, parity, income, race, family history of DM, and personal history of GDM. Pregnancy outcome data was collected based on chart review, and BF status was assessed at V3 via participant questionnaires. Pregnant participants completed an OGTT at all 3 study visits, and nonpregnant participants completed a single OGTT. Participants fasted for at least 8 hours prior to the OGTT. After the fasting blood sample was collected, participants consumed a 75-gram standard OGTT beverage within 5 minutes and blood was subsequently drawn at 30 minutes, 1 hour, and 2 hours after the glucose load.

We used the International Association of the Diabetes in Pregnancy Study Groups’ 2010 criteria to define GDM (fasting glucose ≥92 mg/dL, 1-hour glucose ≥180 mg/dL, 2-hour glucose ≥153 mg/dL) [35] in the first and second trimesters of pregnancy for the purposes of this analysis. Data was collected between 2016 and 2020.

As diet can affect levels of 1,5AG [28, 36, 37], dietary intake information as recorded by the validated self-reported dietary recall ASA24® Dietary Assessment Tool (versions 2016, 2018, or 2020) developed by the National Cancer Institute (Bethesda, MD) was also analyzed [38] (p 24).

Breastfeeding status was collected based on participant self-report at their PP visit. Participants indicated whether they were exclusively BF, combination feeding with breastmilk and formula, or exclusively formula feeding.

We selected pilot study participants from those who attended all 3 study visits using a random number generator. A sample size of n = 50 pregnant/PP individuals and n = 50 nonpregnant individuals was chosen for this pilot study based on the funding resources available and costs of the 1,5AG assay. We shipped frozen plasma samples to the Advanced Research and Diagnostics Laboratory at the University of Minnesota for analysis where 1,5AG levels were measured using the Roche COBAS 8000 chemistry analyzer (Roche Diagnostics, Indianapolis, IN). The stability of 1,5AG testing on frozen samples has been demonstrated based on the work of previous researchers [39].

### Statistical Analysis

We compared baseline demographics of the pregnant cohort to the nonpregnant cohort using parametric statistical tests when appropriate based on normality of the data; for variables that did not appear to be normally distributed, we used nonparametric statistical tests.

To determine the behavior of 1,5AG throughout gestation and PP, we conducted longitudinal analyses of the pregnant cohort with linear mixed models with adjustment for BMI. We then performed an analysis of 1,5AG in pregnant compared to nonpregnant controls at each timepoint with linear regression. Initial analyses were unadjusted, followed by a fully adjusted model including age, BMI, marital status, parity, race/ethnicity, and family history of DM to account for baseline differences between the groups.

We performed descriptive statistics to determine the overall calorie, carbohydrate, and protein intake among both nonpregnant participants as well as pregnant participants at all 3 timepoints based on the returned ASA24 data. Using Spearman correlations, we explored the relationship between 1,5AG and dietary intake in the pregnant and nonpregnant participants.

Using Spearman correlations and linear regression models, we examined the relationship between 1,5AG at the study timepoints and glycemia measures (fasting, 1-hour, 2-hour, mean OGTT glucose values, and A1c). Mean OGTT glucose was calculated based on mean glucose level during the OGTT by averaging the fasting, 1-hour, and 2-hour values. Adjustments in the linear regression models were made to account for gestational age and/or weeks PP.

An association between BF and 1,5AG was explored initially with descriptive statistics. BF status was then stratified into groups including exclusively formula feeding, combination feeding with formula and breastmilk, and exclusively BF. Stratified analyses were then performed to determine the relationship of 1,5AG with BF status using rank-based nonparametric tests (Kruskal-Wallis) followed by post hoc testing (Dunn's test), as well as linear regression models adjusted for fasting glucose. Linear regression models examining the relationship between 1,5AG, BF status, and glycemia were then performed adjusting for weeks PP as described earlier.

In post hoc analyses, the glycemia analyses described previously were also carried out excluding a 1,5AG outlier value that was identified in the course of the main analysis.

Statistical analyses were conducted in STATA IC version 16 (College Station, TX).

## Results

Characteristics of pregnant participants at V1 (n = 50) are presented in Table 1 with comparisons to nonpregnant controls. Pregnant participants were older, less likely to be nulliparous and to have a family history of DM, and more likely to be married. Among the pregnant participants studied, 10 (20%) were diagnosed with GDM in the current pregnancy and 3 (6%) were diagnosed with preeclampsia. In the PP period, 2 (4%) were diagnosed with DM and 1 (2%) was diagnosed with impaired glucose tolerance. The average gestational age at the V1 visit was 12.9 (SD 1.6) weeks’ gestation, at V2 it was 26.2 (SD 1.4) weeks’ gestation, and at V3 it was 11.0 (SD 4.9) weeks PP. Of note, 2 samples were hemolyzed, 1 from a pregnant participant at the V3 timepoint and 1 from a nonpregnant participant.

**Table 1.** Baseline demographics of pregnant and nonpregnant participants at V1

	Nonpregnant (n = 50)	Pregnant (n = 50)	*P*-value
Demographics			
Age (mean, SD)	27.5 (6.5)	32.3 (4.5)	<.001
Race (%)			.07
Hispanic	9 (18)	10 (20)	
White non-Hispanic	21 (42)	31 (62)	
Black non-Hispanic	7 (14)	6 (12)	
Asian	11 (22)	2 (4)	
Other non-Hispanic	2 (4)	1 (2)	
Nulliparous (%)	42 (84)	21 (42)	<.001
BMI (mean, SD)	27.2 (5.4)	29.2 (6.1)	.09
Personal history of GDM (out of parous women) (%)	1 (2)	6 (12)	.05
Family history of diabetes (%)	26 (52)	13 (26)	.008
College graduate	38 (76)	42 (84)	.32
Smoking status (%)			.31
Less than 50 cigarettes in lifetime	45 (90)	39 (78)	
Smoked in past but quit	5 (10)	8 (16)	
Unknown	0 (0)	3 (6)	
Married	6 (12)	37 (74)	<.001
Pregnancy characteristics			
GDM (%)	−	10 (20)	—
Preeclampsia (%)	—	3 (6)	—
Impaired glucose tolerance postpartum (%)	—	1 (2)	—
Diabetes diagnosed postpartum (%)	—	2 (4)	—
Gestational age at V1	—	12.9 (1.6)	—
Gestational age at V2	—	26.2 (1.4)	—
Weeks postpartum at V3	—	10.9 (4.9)	—

Abbreviations: BMI, body mass index; GDM, gestational diabetes mellitus; V1, visit 1 (7-14 weeks); V2, visit 2 (24-30 weeks); V3, visit 3 (6-24 weeks postpartum).

**Figure 1.**
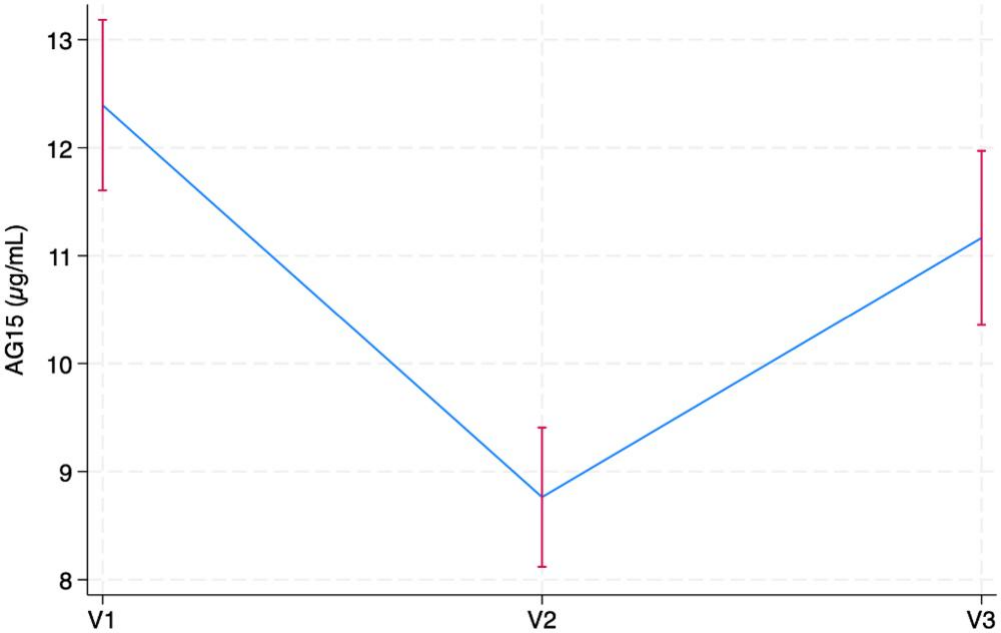
Longitudinal changes in 1,5 anhydroglucitol across gestation and postpartum.

The mean 1,5AG level was 12.4 μg/mL (SD 5.6) at V1, 8.8 μg/mL (SD 4.6) at V2, and 11.2 μg/mL (SD 5.6) at V3. Longitudinal analyses demonstrated that 1,5AG decreased from V1 to V2 (β = −3.6μg/mL [−4.8, −2.5], *P* < .001) and remained decreased PP compared to V1 (β = −1.4μg/mL [−2.5, −0.2], *P* = .018) (Fig. 1). The observed changes in 1,5AG levels were not altered after adjustment for BMI (compared to V1, V2 β = −4.3μg/mL [−5.4, −3.1], *P* < .001, or V3 β = −1.6μg/mL [−2.7, −0.5], *P* = .005).

Pregnant participants were compared to nonpregnant controls. Nonpregnant participants had a mean 1,5AG of 21.9 (7.0 μg/mL). Unadjusted comparisons demonstrated that 1,5AG was lower among pregnant participants across all timepoints (V1: β = −9.5 μg/mL [−11.9, −6.9], *P* < .001; V2: β = −13.1 μg/mL [−15.5, −10.8], *P* < .001; PP β=−10.7 μg/mL [−13.2, −8.1, *P* < .001). These findings were not altered in the fully adjusted model including adjustments for BMI, age, marital status, parity, race/ethnicity, and family history of DM (V1: β = −10.9 μg/mL [−14.4, −7.4], *P* < .001; V2: β = −14.3 μg/mL [−17.6, −11.0], *P* < .001; PP β=−11.2 μg/mL [−14.8, −7.7, *P* < .001).

At V2 and PP, pregnant participants had a higher median kilocalerie intake when compared to nonpregnant participants (*P* = .04 and *P* = .04, respectively). Pregnant participants at V2 had higher mean and median protein intakes when compared to nonpregnant participants (median *P* = .03). No associations between 1,5AG levels and total calorie intake, carbohydrate intake, or protein intake were noted at any of the pregnancy timepoints or in the nonpregnant cohort (Table 2).

**Table 2.** Spearman correlation coefficients between AG15 and diet among nonpregnant and pregnancy timepoints

	Nonpregnant AG15		Pregnant V1 AG15		Pregnant V2 AG15		Postpartum V3 AG15	
	Rho	*P*	Rho	P	Rho	*P*	Rho	*P*
Kilocalorie	−0.21	.18	−0.01	.95	−0.06	.68	−0.17	.26
Carbohydrate	−0.14	.36	0.09	.56	0.06	.70	−0.15	.30
Protein	−0.25	.10	−0.11	.45	−0.16	.27	−0.16	.28

Abbreviations: AG15, 1,5 anhydroglucitol; V1, visit 1 (7-14 weeks); V2, visit 2 (24-30 weeks); V3, visit 3 (6-24 weeks postpartum).

Glycemia analyses revealed no associations between 1,5AG and glycemia at the fasting, 1-hour postload, or 2-hour postload timepoints during pregnancy, nor did we identify associations between 1,5AG and mean OGTT or A1c (Table 3). PP, there was an unexpected positive association between 1,5AG and fasting glucose (Rho = 0.40, *P* = .004). Adjustments for gestational age or weeks PP did not alter these results.

**Table 3.** Spearman correlations between glycemia measures and nonpregnant/pregnancy timepoints

	AG15 nonpregnant (n = 49)		AG15 pregnant V1 (n = 50)		AG15 pregnant V2 (n = 50)		AG15 postpartum V3 (n = 49)	
	Rho	*P*	Rho	*P*	Rho	*P*	Rho	*P*
Fasting	−0.11	.46	0.07	.65	0.19	.1905	0.40	.004
1 hour postload	−0.14	.35	−0.14	.33	−0.15	.30	−0.23	.12
2 hours postload	−0.03	.85	−0.17	.25	−0.05	.75	−0.15	.32
Mean OGTT	−0.08	.57	−0.16	.26	−0.07	.65	0.11	.44
A1c	0.18	.24	0.08	.61	−0.13	.36	−0.002	.99

Abbreviations: AG15, 1,5 anhydroglucitol; OGTT, oral glucose tolerance test; V1, visit 1 (7-14 weeks); V2, visit 2 (24-30 weeks); V3, visit 3 (6-24 weeks postpartum).

### Breastfeeding and 1,5 AG Levels

**Figure 2.**
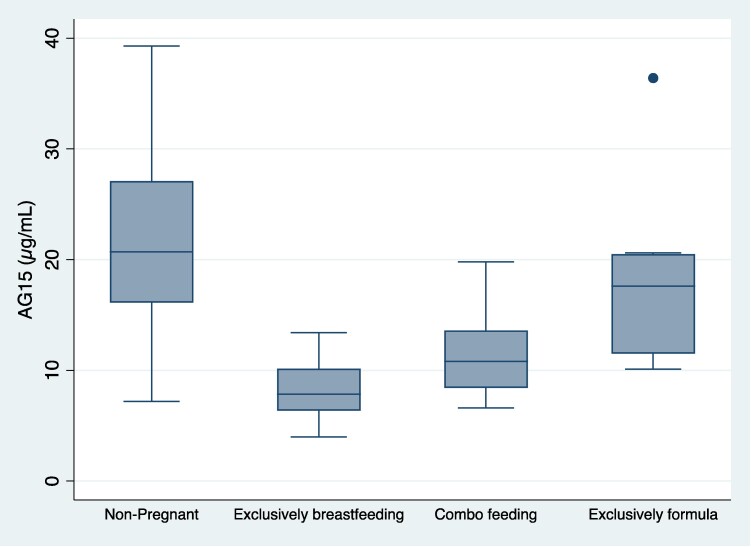
1,5 anhydroglucitol levels stratified by breastfeeding status and among nonpregnant participants.

Twenty-four participants reported exclusive BF, 15 reported combination feeding, and 9 reported exclusive formula feeding. Compared to those exclusively feeding formula, 1,5AG levels were significantly lower in exclusively BF women (β = −8.8 μg/mL [−12.3, −5.3], *P* < .001) and intermediate in women feeding both breastmilk and formula (β = −6.1 μg/mL [−9.8, −2.4], *P* < .001), independent of glycemia and fasting glucose. 1,5AG levels across all 3 groups were notably lower in comparison to nonpregnant participants (Fig. 2). The positive association previously noted between fasting PP glucose and 1,5AG remained positive following adjustments for weeks PP and BF status (β = 0.56 mg/dL, 95% CI 0.06-1.05, *P* = .03). Given the outlier identified, sensitivity analyses were performed to ensure the data in the models remained unchanged when removing this participant. Of note, the outlier's pregnancy was not affected by GDM, and feeding was exclusively formula. Glycemia analyses with the outlier removed were similar to the data previously described.

## Discussion

In this pilot study, we found that 1,5AG decreased throughout gestation and remains lower than baseline levels at the PP timepoint, independent of changes in BMI. We also found that unlike what has been reported outside of pregnancy in people with diabetes, 1,5AG was not inversely associated with any of the measures of glycemia we tested during pregnancy or PP in women without overt diabetes. We did not find an association between dietary intake and serum 1,5AG throughout gestation, PP, or in the nonpregnant cohort. However, serum levels of 1,5AG were lower in women who were exclusively BF compared to those formula feeding their infants.

Prior studies have described the behavior of 1,5AG throughout gestation, though most do not extend this analysis to the PP timepoint. Among those who did evaluate 1,5AG PP, previous work found a return of this marker to baseline [30, 32], which contrasts with our study findings. Our study reaffirms the longitudinal activity of 1,5AG during pregnancy, as well as contributes new findings on the relationship of 1,5 AG with BF to the literature [24, 28, 30, 40, 41].

1,5AG competes with glucose at the level of the renal tubule, and a threshold effect has been demonstrated at approximately 160 to 180 mg/dL of glucose with an expected drop in serum 1,5AG during times of hyperglycemia [42]. As the glomerulus in pregnancy is more permeable to glucose irrespective of glycemia, glycosuria and lower serum levels of 1,5AG may occur irrespective of the presence or absence of dysglycemia [28, 32]. While PP, it is postulated that physiology returns to its nonpregnant state; the exact timing of the return of renal resorption of glucose to “baseline” is unknown, especially among women with some degree of glucosuria during pregnancy [43]. Our findings could be consistent with a slower return of normal renal function as discussed earlier but stand in contrast to prior studies that have demonstrated a return of serum 1,5AG to the normal range within 30 days PP. Tetsuo et al [32] observed 543 pregnant patients with normal glycemia and 75 with DM or GDM throughout gestation and extending through 30 days PP and found that among participants with normal glycemia in pregnancy as measured by serum glucose, 1,5AG returned to normal nonpregnant levels within 30 days PP. While this study was large and measured 1,5AG throughout pregnancy and PP, they did not adjust their findings for baseline characteristics, nor did they collect data regarding or perform adjusted analyses for PP factors including BMI or BF status. Our findings demonstrate not only that participant 1,5AG levels do not increase back to the nonpregnant state within a mean of 11 weeks PP but also a likely mechanism of excretion of 1,5AG into breastmilk. Studies of breastmilk composition have reported findings of 1,5AG in human breastmilk spanning from 1 to 6 months after birth [44, 45]. Our findings, combined with these others, suggest that 1,5AG excretion occurs not only through the renal system but also through breastmilk. Our study should prompt further investigation into the PP physiology. The literature lacks significant and meaningful data with respect to PP physiology, as well as a lack of data with respect to the interaction between BF and various aspects of PP health, including glycemia.

Despite the promise of a biomarker like 1,5AG, our findings do not support its use over the OGTT in pregnancy or in the PP period as a screening tool for dysglycemia or diabetes. The physiology of the PP period remains incompletely understood as evidenced by the lack of return of 1,5AG levels to levels in nonpregnant and non-PP individuals, as well as the lack of predictability and utility of A1c in this period [16-20]. Our study continues to demonstrate the need to identify reliable biomarkers that can be used for screening for PP dysglycemia, as opposed to continued reliance on the OGTT, which has suboptimal completion rates.

Strengths of our study include its longitudinal design, the utilization of a cohort at high risk for GDM, and the inclusion of A1c measurements in the analysis. We also note several limitations. First, OGTT glucose levels capture glycemia at a moment in time in response to a glucose load and do not necessarily correlate with glycemia during daily life, whereas 1,5AG is more likely to reflect mean glycemia in the preceding days to weeks. Second, our sample size was limited; however, if an association between 1,5AG and glycemia was not identified with 50 participants, it is unlikely to be meaningful on an individual patient level. Lastly, our study population was at high risk for dysglycemia and may not be generalizable across the entire population. Despite this, given the high-risk population in this cohort, our findings may be the most relevant to the population of patients most likely to undergo early pregnancy screening, to be diagnosed with GDM, and therefore to require PP glycemic assessment.

In conclusion, we have demonstrated that 1,5AG fails to correlate with glycemia throughout gestation or in the PP period in those without overt diabetes. We have also demonstrated a plausible excretion pathway of 1,5AG through breastmilk, leading to lower levels in the PP period. Further research should be directed to identify other biomarkers that could yield reliable results with respect to pregnant and PP glycemic status without the burden of an OGTT. Additionally, future efforts should be directed to better understand PP physiology as well as the relationship of BF to physiology and the processing of various biomarkers.

## Data Availability

Some or all datasets generated during and/or analyzed during the current study are not publicly available but are available from the corresponding author on reasonable request.

## Prior Presentation

This work was presented as a poster at the Society for Maternal-Fetal Medicine 42nd Annual Meeting, February 2022. Virtual conference.
